# Karyotype diversity and patterns of chromosomal evolution in *Eigenmannia* (Teleostei, Gymnotiformes, Sternopygidae)

**DOI:** 10.3897/CompCytogen.v8i4.8396

**Published:** 2014-11-17

**Authors:** Viviani França Sene, José Carlos Pansonato-Alves, Ricardo Utsunomia, Claudio Oliveira, Fausto Foresti

**Affiliations:** 1 Laboratório de Biologia e Genética de Peixes, Instituto de Biociências de Botucatu, Universidade Estadual Paulista (UNESP), Departamento de Morfologia, Distrito de Rubião Junior, Botucatu, São Paulo, Brazil. CEP: 18618-970

**Keywords:** Fish comparative cytogenetics, electric fishes, chromosome banding, rDNA variability, sex chromosomes

## Abstract

Conventional (Giemsa, C-banding, Ag – NORs) and molecular [5S rDNA, 18S rDNA, (TTAGGG)_n_] cytogenetic techniques were employed to study six species of the genus *Eigenmannia* Jordan & Evermann, 1896. They exhibited diploid chromosome numbers ranging from 2n=28 (*Eigenmannia* sp.1) to 2n=38 (*Eigenmannia virescens* (Valenciennes, 1836)). The C-banding results revealed that species with the lowest 2n have less heterochromatin content and that morphologically differentiated sex chromosomes observed in two species showed distinct patterns of heterochromatin. While the X_1_, X_2_ and Y-chromosomes of *Eigenmannia* sp.2 showed only centromeric heterochromatin, the XY sex chromosomes of *Eigenmannia virescens* possessed large heterochromatic blocks in the terminal position, particularly on the X chromosome. The nucleolus organizer regions (NORs) were located in different positions when compared to the 5S rDNA sites. Additionally, the presence of minor ribosomal gene sites on the sex chromosome pair of *Eigenmannia virescens* represented a new type of the sex chromosomes in this group. The telomeric probe (TTAGGG)_n_ hybridized to the terminal portion of all chromosomes in all species examined however, interstitial telomeric sites were found in the metacentric pair No. 2 in *Eigenmannia* sp.1. The analyzes confirmed some hypotheses about karyotype evolution in the genus *Eigenmannia*, and brought new information about the distribution of the genetic material in the chromosomes of the samples analyzed providing new insights for understanding the process differentiation in the genomes of species under study.

## Introduction

Fishes of the Gymnotiformes order, known as “electric knifefishes”, constitute an endemic group in Neotropical freshwaters (Albert and Crampton 2003). This group comprises more than 100 species classified into five families, namely Gymnotidae, Rhamphichthyidae, Hypopomidae, Sternopygidae, and Apteronotidae (Reis et al. 2003). The genus *Eigenmannia* Jordan & Evermann, 1896, family Sternopygidae, is represented by eight widely distributed species (Albert 2001). However, the actual taxonomic diversity of this genus is still unclear, mainly because presently recognized species very likely include other undescribed species, i.e. they represent catch-all taxa.

Available cytogenetic data for *Eigenmannia* species show a remarkable karyotype diversification, including the occurrence of distinct diploid chromosome numbers, ranging from 2n = 28 to 38 chromosomes, and several sex chromosome systems (Almeida Toledo and Foresti 2001; Henning et al. 2008; Silva et al. 2009). However, studies on the distribution of repetitive sequences are scarce and still restricted to chromosomes of a single species – *Eigenmannia virescens* (Valenciennes, 1836) (Silva et al. 2009).

Remarkably, the distribution of repetitive DNAs in the genomes of *Gymnotus* Linnaeus, 1758, another genus within the order Gymnotiformes, is well known and showed that individual multigene families may be extremely variable (e.g. 5S rDNA) or conserved (U2 snDNA and 18S rDNA) at the species level (Scacchetti et al. 2011, 2012; Milhomem et al. 2013; Utsunomia et al. 2014). Therefore, the cytogenetic mapping may be a valuable tool to provide insights into the evolutionary relationships among close species and allow a better comprehension of the distribution and organization of repetitive sequences in the genomes of several species.

The main aim of the present study was to increase the knowledge about karyotype structure of six different *Eigenmannia* species. Additionally, the chromosomal location of telomeric repeats and ribosomal genes was revealed by fluorescence *in situ* hybridization (FISH).

## Materials and Methods

**Figure 1.**
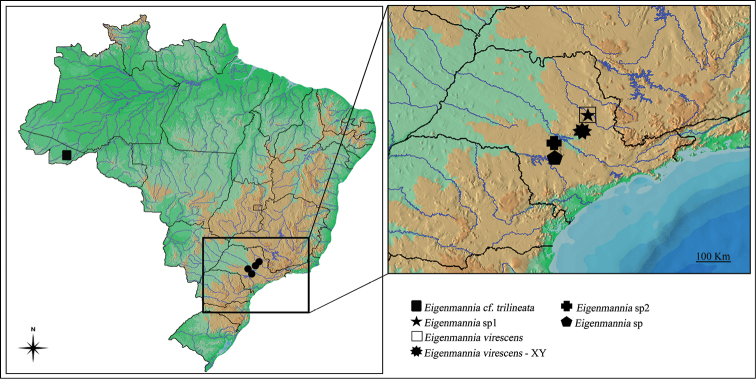
Map of Brazil showing the collection sites of the *Eigenmannia* populations analyzed.

Fishes were collected in distinct river basins (Table 1, Fig. 1). The fishes were collected in accordance with Brazilian environmental protection legislation (Collection Permission MMA / IBAMA / SISBIO – number 3245) and the procedures for collection, maintenance and analysis of fish samples were performed with the international protocols on animal experimentation followed by the Universidade Estadual Paulista. The sampled individuals analyzed were fixed in 10% formaldehyde, preserved in 70% ethanol and deposited in the collection of the Laboratory of Fish Biology and Genetics (LBP), UNESP, Botucatu, São Paulo, Brazil under the identification number 521 LBP (Table 1).

Mitotic chromosomes were obtained from cell suspensions of the anterior kidney (Foresti et al. 1981). Nucleolus organizer regions (NORs) were identified by silver (Ag) nitrate staining (Howell and Black 1980), and C-banding patterns were obtained following the protocol by Sumner (1972). Genomic DNA was obtained from muscle using the Wizard Genomic DNA Purification Kit (Promega) according to the manufacturer’s instructions. Fluorescence *in situ* hybridization (FISH) was accomplished according to Pinkel et al. (1986).

(TTAGGG)n, major (18S rDNA) and minor (5S rDNA) ribosomal probes were isolated from the genome of *Eigenmannia* sp. 2 by PCR using previously described primers (White et al. 1990; Ijdo et al. 1991; Pendás et al. 1994). The 18S rDNA sequences were labeled with Digoxigenin-11-dUTP (Roche Applied Science), and the 5S rDNA and (TTAGGG)n probes probe were labeled with biotin-16-dUTP (Roche Applied Science). Detection of hybridization signals was performed using anti-digoxigenin-rhodamine (Roche Applied Science) and avidin-FITC.

The chromosomes were cut using Adobe Photoshop version 11.0 software - Adobe Systems and organized were arranged in putative homologous pairs in the karyotypes, and classified as metacentric (m), submetacentric (sm), subtelocentric (st), and acrocentric (a) (Levan et al. 1964) and disposed in order of decreasing size in two groups consisting of metacentric-submetacentric and subtelocentric-acrocentric chromosomes.

**Table 1.** Individuals of *Eigenmannia* species analyzed, diploid chromosome number 2n, collecting localities. LBP – deposit voucher number at the fish collection of the Laboratório de Biologia e Genética de Peixes, Instituto de Biociências de Botucatu, UNESP.

Species	2n	Materials	Sample localities	Coordinates	LBP
*Eigenmannia virescens*	38	11♀ 09♂	Mogi-Guaçu river, Araras, São Paulo	S21°56'35", W47°23'04"	12307
*Eigenmannia virescens* -XY	38-XY	01♀ 01♂	Ribeirão Claro stream, Rio Claro, São Paulo	S22° 21'28.3", W47°30'51.4"	12308
Eigenmannia cf. trilineata	34	08♀ 06♂	Acre river, Rio Branco, Acre	S09°56'16.6", W67°52'43.6"	12303
*Eigenmannia* sp.	36	01♀ 01♂	Hortelã river, Botucatu, São Paulo	S22°55'23.22", W48°32'40.46"	12304
*Eigenmannia* sp.1	28	05♀ 06♂	Mogi-Guaçu river, Araras, São Paulo	S21°56'35", W47°23'04"	12305
*Eigenmannia* sp.2	31/32 -X_1_X_1_X_2_X_2_-X_1_X_2_Y	10♀ 08♂	Araquá river, Botucatu, São Paulo	S22°47'13", W48°28'89"	12306

## Results

**Figure 2.**
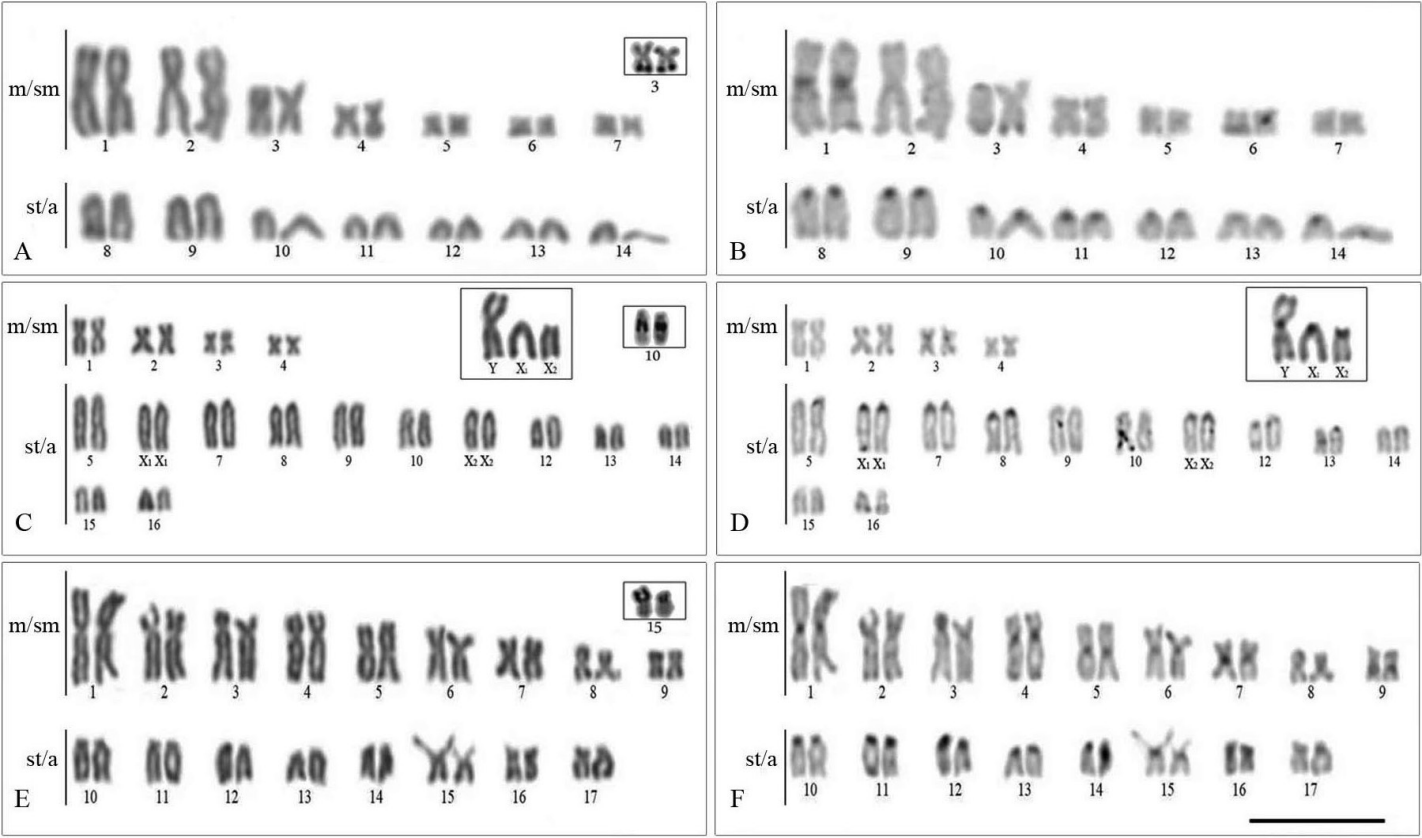
Karyotypes of *Eigenmannia* sp.1 (**a, b**), *Eigenmannia* sp.2 (**c, d**), Eigenmannia cf. trilineata (**e, f**), arranged from Giemsa stained (**a, c, e**) and C-banded chromosomes (**b, d, f**). Inset shows the Ag-NOR-bearing chromosomes (**a, c, e**). Inset shows the male sex chromosomes of *Eigenmannia* sp.2 (**c, d**). Bar =10 µm.

**Figure 3.**
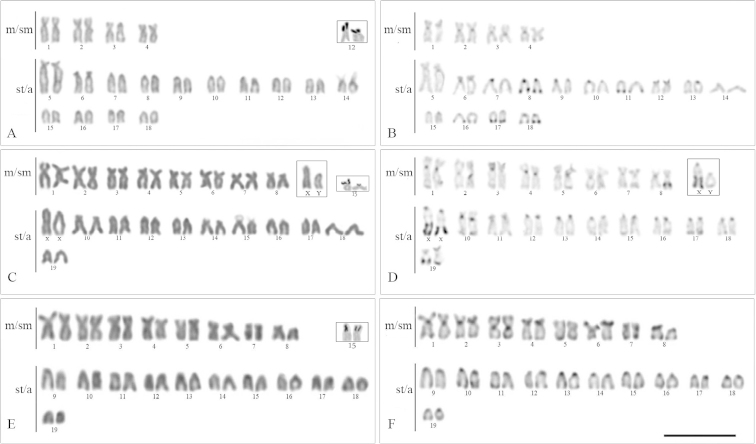
Karyotypes of *Eigenmannia* sp. (**a, b**), *Eigenmannia virescens* - XY (**c, d**), *Eigenmannia virescens* (**e, f**), arranged from Giemsa stained (**a, c, e**) and C-banded chromosomes (**b, d, f**). Inset shows the Ag-NOR-bearing chromosomes (**a, c, e**). Inset shows the male sex chromosomes of *Eigenmannia virescens* - XY (**c, d**). Bar =10 µm.

Diploid chromosome numbers ranged from 2n=28 chromosomes in *Eigenmannia* sp.1 to 2n=38 in *Eigenmannia virescens* (Table 1). Moreover, the individuals of *Eigenmannia* sp. 2 from the Araquá River had a multiple sex chromosome system of X_1_X_1_X_2_X_2_/X_1_X_2_Y type.(Fig. 2C–D), while *Eigenmannia virescens* had an XY sex chromosome system (Fig. 3C–D). The constitutive heterochromatin was preferentially located in the pericentromeric regions of all chromosomes of the analyzed species. Additionally, a conspicuous accumulation of heterochromatin in the X chromosome of *Eigenmannia virescens* was also observed (Fig. 3D). Ag-NORs were located in a single chromosome pair in all species.

**Figure 4.**
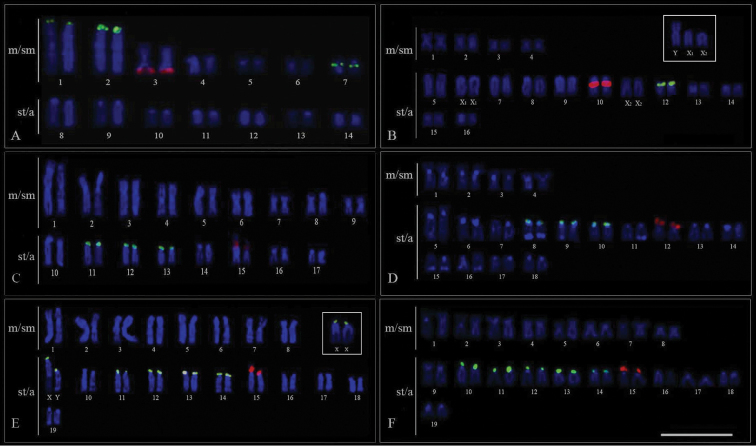
Karyotypes of the analyzed *Eigenmannia* species after FISH with 5S (green) and 18S (red) ribosomal probes and counterstained with DAPI. **a** *Eigenmannia* sp.1 **b** *Eigenmannia* sp.2 **c** Eigenmannia cf. trilineata **d** *Eigenmannia* sp. **e** *Eigenmannia virescens* –XY **f** *E. virescens.* Inset shows the male sex chromosomes of *Eigenmannia* sp.2 (**b**) and *Eigenmannia virescens* – XY (**e**). Bar =10 µ.

FISH analyses using 18S rDNA probes confirmed the Ag-NOR sites (Fig. 4). Conversely, the minor ribosomal sites presented a distinct number of sites per genome, from 2 to 10, in different species. However, the position of these sites, mostly located in the centromeric region of st/a chromosomes, was conserved, except for *Eigenmannia* sp.1 (Fig. 4a–f).

**Figure 5.**
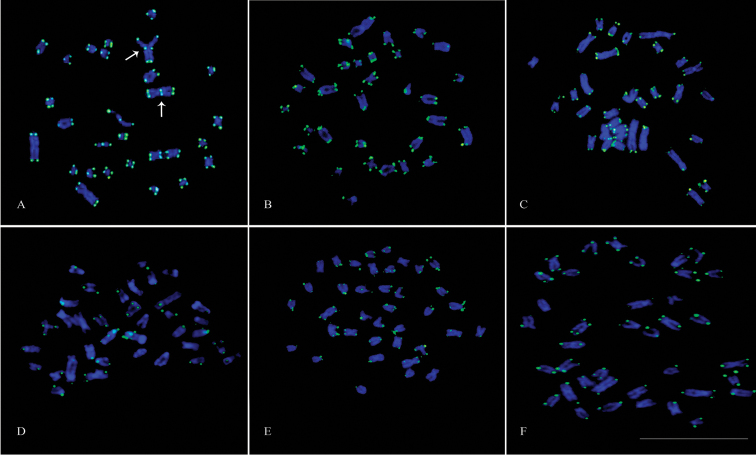
Mitotic metaphase chromosomes of *Eigenmannia* species hybridized with telomeric probes. **a** *Eigenmannia* sp.1 note interstitial telomeric sites (ITS) in chromosome pair 2 **b** *Eigenmannia* sp.2 **c** *Eigenmannia* prope *trilineata* **d** *Eigenmannia* sp. **e** *Eigenmannia virescens* **f** *Eigenmannia virescens*–XY. Bar =10 µm.

Telomeric probes revealed hybridization signals in the terminal position of almost all chromosomes in all species examined (Fig. 5). Additionally, interstitial sites were observed in the m pair 2 of *Eigenmannia* sp.1 (Fig. 5a).

## Discussion

The genus *Eigenmannia* is a fish group that shows complex morphological patterns. Cytogenetic studies performed in this group revealed great karyotype diversity among species and populations, including the occurrence of karyomorphs with different heteromorphic sex chromosomes (Silva et al. 2009; Henning et al. 2010). Considering their territorial behavior the fixation of different karyotypes via chromosomal rearrangements could be promoted by reproductive isolation and low levels of interchange among individuals from different small rivers (Moysés et al. 2010).

The significant chromosomal variability observed in the present study is consistent with previous studies of this genus (Almeida-Toledo et al. 1985; Almeida-Toledo et al.1996) and highlights the importance of cytogenetics as a tool in the study of relationships among knifefish representatives. Since the 2n is remarkably diversified in *Eigenmannia*, it has been suggested that chromosomal fusions and fissions are mechanisms that played an important role in the karyotype diversification within this group (Almeida-Toledo et al. 2000, 2001, 2002; Almeida-Toledo and Foresti 2001; Henning et al. 2008). FISH analyses corroborated this hypothesis and indicated that pair No. 2 of *Eigenmannia* sp.1 probably originated via a centric fusion, due to its decreased number of st/a chromosomes when compared to other species.

In a pioneer study, Milhomem et al. (2013) showed that despite the occurrence of a high karyotype variability in *Gymnotus* species, the NOR-bearing chromosomes are homologous in distinct species. Our analyses documented that a similar situation may occur in *Eigenmannia*, since the NOR-bearing chromosomes of *Eigenmannia* sp, *Eigenmannia virescens* and Eigenmannia cf. trilineata López & Castello, 1966 are very similar and possibly homologous among them, bearing the major ribosomal sites in the terminal position on the p arms. However, in the species with lower 2n, the location of these sites is species-specific, indicating that the NOR-bearing chromosomes might have been involved in chromosomal rearrangements during their differentiation process. *Eigenmannia* sp.2 is the only species showing NORs located at the interstitial position, conceivably indicating that pair No. 10 of this species may have arisen through fusion events involving ancestral chromosomes carrying ribosomal sequences.

The chromosomal location of 5S rRNA sites was described for the first time in *Eigenmannia* and showed that unlike 18S rDNA, the minor ribosomal sites present an extensive evolutionary variation in this group. A similar scenario was also observed in *Gymnotus*, in which chromosomal location of 5S rDNA is diversified among different species, probably because of its association with transposable elements (da Silva et al. 2011; Scacchetti et al. 2011, 2012). However, the chromosomal location of these sites does not seem to have changed in a short span of time in *Eigenmannia virescens* because various cytotypes of 5S rDNA sites diverged recently (<0.6mya) (Henning et al. 2010). Actually, the ribosomal sites 5S are probably conserved in the same five pairs (Fig. 4E–F), including the XX/XY sex chromosomes. Such organization implies that these sex chromosomes are not yet well differentiated, with the accumulation of heterochromatin in the X being the primary cause of diversification of the sex chromosomes, as suggested by previous studies (Henning et al. 2010).

**Figure 6.**
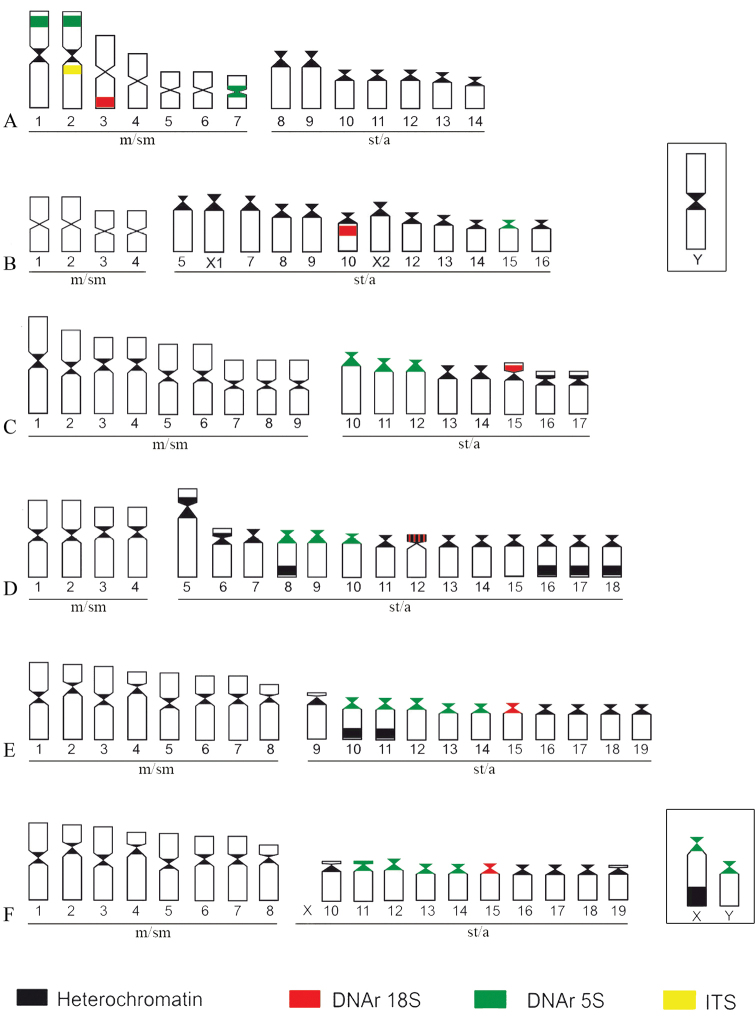
Ideograms showing C-heterochromatin and hybridization patterns described in this study for: **a** *Eigenmannia* sp.1 **b** *Eigenmannia* sp.2 **c** Eigenmannia cf. trilineata **d** *Eigenmannia* sp. **e** *Eigenmannia virescens* **f** *Eigenmannia virescens*–XY.

The present study confirmed the high diversity in the chromosome structure among the representatives of *Eigenmannia*. It also corroborates the occurrence of sex-linked chromosome polymorphisms, indicating the presence of extensive chromosomal rearrangements with *Eigenmannia* species at the genome macro and microstructure levels, of the genetic material, providing new insight for understanding the contributing evidently to speciation processes. (Fig. 6).
